# P-1642. The Devil's in the details: Developing metrics for discharge antibiotic stewardship

**DOI:** 10.1093/ofid/ofae631.1808

**Published:** 2025-01-29

**Authors:** Marten R Hawkins, Ritika Prasad, Leah Mische, Esther Esadah, Emily Mui, Samaneh Pourali, David R Ha, Marisa Holubar

**Affiliations:** University of Michigan, Stanford, California; Stanford Health Care, Stanford, California; Stanford University Health Care, Palo Alto, California; Children's National Hospital, Washington, District of Columbia; Stanford Health Care, Stanford, California; Stanford Health Care, Stanford, California; Stanford Health Care, Stanford, California; Stanford University School of Medicine, Stanford, CA

## Abstract

**Background:**

International Classification of Diseases, Tenth Revision codes (ICD-10s) are commonly used to identify antibiotic indications but may not reflect clinician intent. We investigated concordance of different antibiotic indication sources in the electronic health record (EHR) with clinician intent upon discharge.

**Figure 1:**
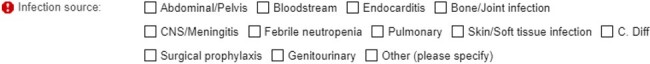
Indication choices for inpatient antibiotic orders

**Methods:**

In this quality improvement project, we included prescriptions for patients discharged on oral antibacterials from January 2022-March 2023, including patients with > 1 admission. We excluded those with >90 days duration and those for dapsone or rifaximin. We randomly selected 100 prescriptions and performed manual chart review to obtain the discharge summary antibiotic indications which we categorized into syndromes (Table 1.) We compared discharge indication with other EHR indication sources: last inpatient antibiotic order indication (is required, Figure 1), ICD-10s from separate fields ( “principle”, “encounter”, “billing”) and “composite ICD-10” which included all ICD-10s from any field. We compared each indication source with the associated discharge indication (considered most accurate) and report concordance.

**Table 1:**
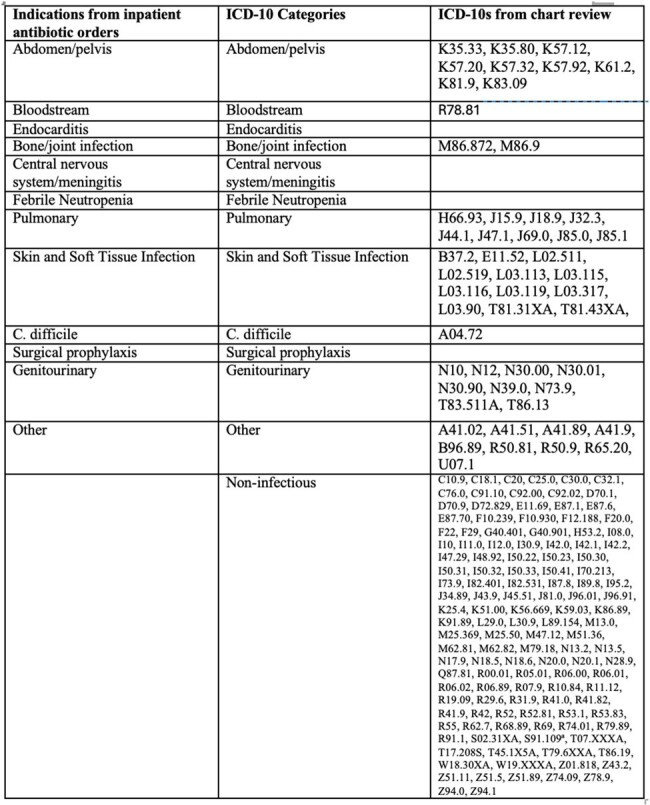
Syndrome Categories and associated ICD-10 codes

**Results:**

We included 3,088 prescriptions and excluded 364. Of the 100 encounters reviewed, 62% patients were discharged from medicine services and 46% had a trainee involved in discharge. These encounters were associated with 184 unique ICD-10s and 132 (72%) representing non-infectious diagnoses; the most common infectious diagnosis was skin and soft tissue infections (12 (7%)) (Table 2.) Overall, 37% (30/100) of prescriptions had ICD-10s that were categorized only as non-infectious. Clinicians most commonly intended discharge antibiotics for genitourinary indications (32/108, 30%) (Table 2). Last inpatient antibiotic order indication matched clinician intent in 91% of cases, while composite ICD-10 matched clinician intent in 47% of cases. Any single ICD-10 field (principle, billing, encounter) matched clinician intent in only 1/3 of cases (Table 3.)

**Table 2:**
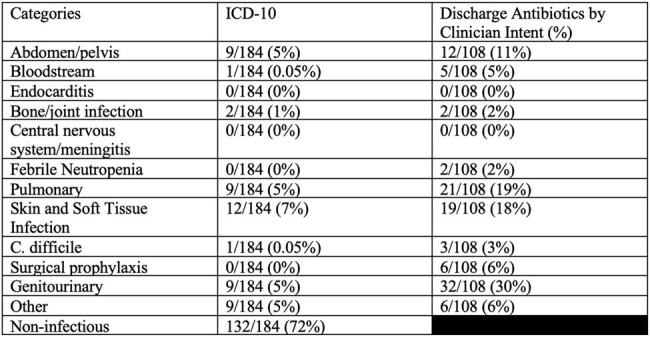
ICD-10 codes and Clinician Intent by Syndrome

Data from 100 chart reviews. A total of 184 unique ICD-10 were collected from 100 prescriptions. 8 discharge summaries included two indications.

**Conclusion:**

Compared to ICD-10s, last inpatient antibiotic order indication more accurately matched clinician intent for antibacterial discharge prescriptions. This may be a useful and simple way to facilitate discharge prescription stewardship.

**Table 3:**
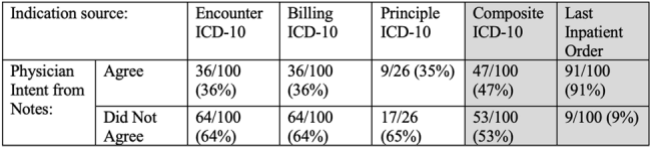
Agreement of different indication sources from EHR with ICD-10 code

“Composite ICD-10” includes all ICD-10s from “principle”, “encounter”, “billing” fields. And was considered to match clinical intent if any ICD-10 category was concordant with clinical intent.

**Disclosures:**

**All Authors**: No reported disclosures

